# Crystal structure of *catena*-poly[[aqua(2,2′:6′,2′′-terpyridine-κ^3^ *N*,*N*′,*N*′′)cobalt(II)]-μ-cyanido-κ^2^ *N*:*C*-[dicyanidoplatinum(II)]-μ-cyanido-κ^2^ *C*:*N*]

**DOI:** 10.1107/S1600536814017425

**Published:** 2014-08-06

**Authors:** Frankie White, Richard E. Sykora

**Affiliations:** a University of South Alabama, Department of Chemistry, Mobile, AL 36688-0002, USA

**Keywords:** crystal structure, cobalt/platinum complex, coordination polymer, hydrogen bonding, π–π stacking

## Abstract

The title compound, [Co(C_15_H_11_N_3_)(H_2_O){Pt(CN)_4_}]_*n*_, is a one-dimensional coordination polymer formed under hydro­thermal reaction conditions. The Co^II^ site has sixfold coordination with a distorted octa­hedral geometry, while the Pt^II^ ion is coordinated by four cyanide groups in an almost regular square-planar geometry. The compound contains twofold rotation symmetry about its Co^II^ ion, the water molecule and the terpyridine ligand, and the Pt^II^ atom resides on an inversion center. *trans*-Bridging by the tetra­cyanidoplatinate(II) anions links the Co^II^ cations, forming chains parallel to [-101]. Additionally, each Co^II^ atom is coordin­ated by one water mol­ecule and one tridentate 2,2′:6′,2′′-terpyridine ligand. O—H⋯N hydrogen-bonding inter­actions are found between adjacent chains and help to consolidate the crystal packing. In addition, relatively weak π–π stacking inter­actions exist between the terpyridine ligands of adjacent chains [inter­planar distance = 3.464 (7) Å]. No Pt⋯Pt inter­actions are observed in the structure.

## Related literature   

For structural studies on related coordination compounds, see: Maynard *et al.* (2008 ▶); Smith *et al.* (2012 ▶); Guo *et al.* (2012 ▶); Kobayashi *et al.* (2013 ▶). For characterization of tetra­cyanido­platinate compounds, see: Gliemann & Yersin (1985 ▶).
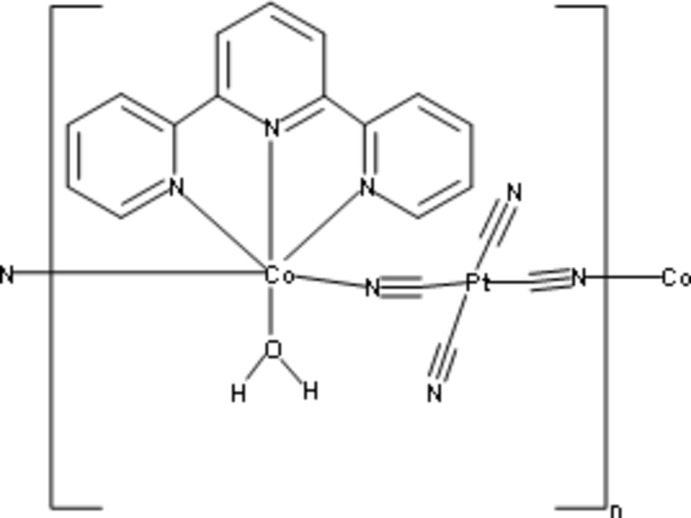


## Experimental   

### Crystal data  

[Co(C_15_H_11_N_3_)(H_2_O){Pt(CN)_4_}]
*M* *_r_* = 609.38
Monoclinic, 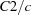

*a* = 15.7272 (7) Å
*b* = 11.5164 (5) Å
*c* = 11.4048 (5) Å
β = 99.005 (4)°
*V* = 2040.20 (16) Å^3^
*Z* = 4
Mo *K*α radiation
μ = 7.69 mm^−1^
*T* = 180 K
0.56 × 0.10 × 0.08 mm

### Data collection  

Agilent Xcalibur Eos diffractometer
Absorption correction: multi-scan (*CrysAlis PRO*; Agilent, 2014 ▶) *T*_min_ = 0.264, *T*_max_ = 1.000
4788 measured reflections
1861 independent reflections
1262 reflections with *I* > 2σ(*I*)
*R*_int_ = 0.041

### Refinement  

*R*[*F* ^2^ > 2σ(*F* ^2^)] = 0.034
*wR*(*F* ^2^) = 0.091
*S* = 1.02
1861 reflections
138 parameters
4 restraints
H atoms treated by a mixture of independent and constrained refinement
Δρ_max_ = 1.42 e Å^−3^
Δρ_min_ = −1.52 e Å^−3^

### 

Data collection: *CrysAlis PRO* (Agilent, 2014 ▶); cell refinement: *CrysAlis PRO*; data reduction: *CrysAlis PRO*; program(s) used to solve structure: *SHELXS97* (Sheldrick, 2008 ▶); program(s) used to refine structure: *SHELXL97* (Sheldrick, 2008 ▶); molecular graphics: *OLEX2* (Dolomanov *et al.*, 2009 ▶); software used to prepare material for publication: *OLEX2* and *publCIF* (Westrip, 2010 ▶).

## Supplementary Material

Crystal structure: contains datablock(s) I, New_Global_Publ_Block. DOI: 10.1107/S1600536814017425/hg5401sup1.cif

Structure factors: contains datablock(s) I. DOI: 10.1107/S1600536814017425/hg5401Isup2.hkl

**Fig. 1.**
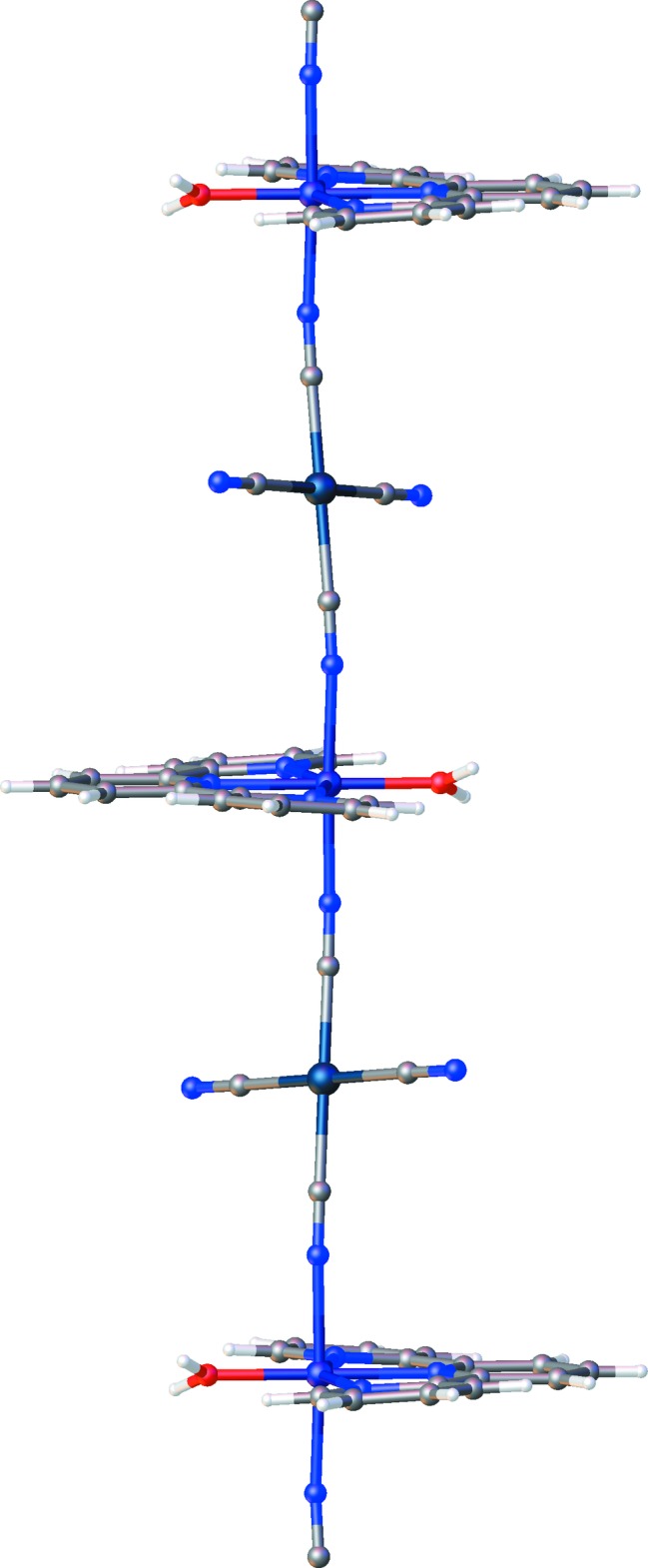
A ball-and-stick representation of the one-dimensional chains in (I).

. DOI: 10.1107/S1600536814017425/hg5401fig1.tif
A ball-and-stick representation of the one-dimensional chains in (I).

**Fig. 2.**
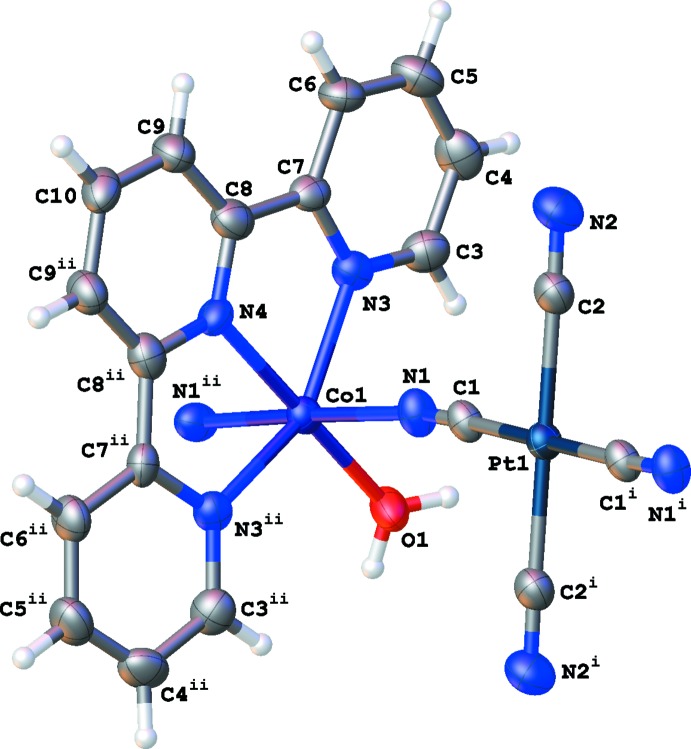
A thermal ellipsoid plot of (I) with the atom-numbering scheme. Displacement ellipsoids for non-hydrogen atoms are drawn at the 50% probability level. H-atoms are shown as spheres of arbitrary size. Symmetry codes: (i) -x + 3/2, -y + 1/2, -z; (ii) -x + 1, y, -z + 1/2.

Click here for additional data file.x y z x y z . DOI: 10.1107/S1600536814017425/hg5401fig2.tif
A thermal ellipsoid plot of (I) with the atom-numbering scheme. Displacement ellipsoids for non-hydrogen atoms are drawn at the 50% probability level. H-atoms are shown as spheres of arbitrary size. Symmetry codes: (i) −*x* + 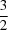, −*y* + 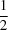, −*z*; (ii) −*x* + 1, *y*, −*z* + 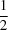.

CCDC reference: 1016798

Additional supporting information:  crystallographic information; 3D view; checkCIF report

## Figures and Tables

**Table 1:** Hydrogen-bond geometry (Å, °)

*D*—H⋯*A*	*D*—H	H⋯*A*	*D*⋯*A*	*D*—H⋯*A*
O1—H1⋯N2^i^	0.85 (1)	1.93 (2)	2.764 (8)	168 (9)

Symmetry code: (i) 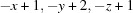.

## supplementary crystallographic information

### S1. Comment

The title compound, (I), results from ongoing research concerning the synthesis of bimetallic coordination polymers containing cyano­metallates. Compound I is similar to several previously reported compounds in that it contains one-dimensional [Co(C_15_H_11_N_3_)(H_2_O)(Pt(CN)_4_)]_n_ chains reminiscent of those found in [Co(C_15_H_11_N_3_)(Pt(SCN)_4_] (Kobayashi *et al.*, 2013). Several related lanthanide coordination polymers Ln(C_15_H_11_N_3_)(H_2_O)_2_(NO_3_)[Pt(CN)_4_].CH_3_CN (Ln = Eu (Maynard *et al.*, 2008) or Ln = Tb (Smith *et al.*, 2012)) with tetra­cyano­platinate(II) are also known. The major structural differences between these latter structure types can be attributed to the higher coordination number that the Ln^3+^ ions typically adopt, relative to Co^2+^ (Guo *et al.*, 2012).

The neutral, one-dimensional [Co(C_15_H_11_N_3_)(H_2_O)(Pt(CN)_4_)] chains in the structure of I are illustrated in Figure 1 and a thermal ellipsoid plot of the local metal ion environments are illustrated in Figure 2. The chains are formed by the linkage of the Co^2+^ cations by *trans*-bridging tetra­cyano­platinate anions. These are reminiscent of the chains found in the bimetallic compound [Mn(C_15_H_11_N_3_)(Pt(SCN)_4_] (Kobayashi, *et al.*, 2013), where similar bridging of the Mn^2+^ ion by the [(Pt(SCN)_4_] anions are observed. The coordination of the Co site is six-fold and can be described as a distorted [CoON_5_] o­cta­hedron while the Pt site has a four-fold coordination in a nearly regular square planar geometry. The compound contains two fold symmetry about its Co^II^ ion and the Pt^II^ resides on an inversion center. The five nitro­gen atoms in the inner sphere of the Co^2+^ cations result from the coordination of one tridentate terpyridine ligand and two N-bound TCP anions while the oxygen atom is a result of one coordinated water molecule. The Co—N, Co—O, and Pt—C bond distances are not extraordinary.

The predominant inter-chain features in I include inter-chain hydrogen bonding inter­actions, see hydrogen bond table, and also weak π-stacking inter­actions (3.464 (7) Å). Also worth noting is the orientation of the coordinated tpy molecules in the one-dimensional chains; viewing parallel to the chain reveals that these molecules are located on alternating sides of the chains. A similar situation also occurs in [Eu(C_15_H_11_N_3_)(H_2_O)_2_(NO_3_)Pt(CN)_4_].CH_3_CN (Maynard *et al.*, 2008) while [Tb(C_15_H_11_N_3_)(H_2_O)_2_(NO_3_)Pt(CN)_4_].3.5H_2_O (Smith, *et al.*, 2012) contains one-dimensional chains where all of the terpyridine molecules reside on a single side of the chain. There are not any platinophilic (Pt···Pt) inter­actions in this compound as observed in many previous tetra­cyano­platinate salts (Gliemann & Yersin, 1985).

### S2. Synthesis and Crystallization

The title compound was synthesized by first mixing aqueous solutions of 0.05 M CoClO_4_ and 0.05 M K_2_[Pt(CN)_4_] (500 µL each). A pink precipitate was immediately formed which was then separated from the mother liquor by centrifugation followed by decantation. The resultant pink solid was placed in an oven at 110 °C for approximately one hour during which time it underwent a color transformation from pink to violet purple. A few milligrams of the powder was placed into a 23 mL teflon-lined Parr reaction vessel with 500 µL of deionized water. The reaction vessel was then heated in a box oven at 110 °C for 72 hours. During this process, impregnated 2,2':6',2"-terpyridine leached out of the teflon liner into the reaction. Once the reaction vessel had cooled pink needle-shaped single crystals of the title compound were isolated.

### S3. Refinement

Crystal data, data collection and structure refinement details are summarized in Table 1. H-atoms were placed in calculated positions and allowed to ride during subsequent refinement, with *U*_iso_(H) = 1.2*U*_eq_(C) and C—H distances of 0.93 Å for ring hydrogens and *U*_iso_(H) = 1.5*U*_eq_(O) and O—H distances of 0.85 Å for hydrogen atoms of the water.

### Crystal data

**Table:**

[CoPt(CN)_4_(C_15_H_11_N_3_)(H_2_O)]	*F*(000) = 1156
*M**_r_* = 609.38	*D*_x_ = 1.984 Mg m^−^^3^
Monoclinic, *C*2/*c*	Mo *K*α radiation, λ = 0.71073 Å
*a* = 15.7272 (7) Å	Cell parameters from 1258 reflections
*b* = 11.5164 (5) Å	θ = 4.0–28.1°
*c* = 11.4048 (5) Å	µ = 7.69 mm^−^^1^
β = 99.005 (4)°	*T* = 180 K
*V* = 2040.20 (16) Å^3^	Needle, clear pink
*Z* = 4	0.56 × 0.10 × 0.08 mm

### Data collection

**Table:**

Agilent Xcalibur Eos diffractometer	1861 independent reflections
Radiation source: Enhance (Mo) X-ray Source	1262 reflections with *I* > 2σ(*I*)
Graphite monochromator	*R*_int_ = 0.041
Detector resolution: 16.0514 pixels mm^-1^	θ_max_ = 25.3°, θ_min_ = 3.5°
ω scans	*h* = −18→18
Absorption correction: multi-scan (*CrysAlis PRO*; Agilent, 2014)	*k* = −13→13
*T*_min_ = 0.264, *T*_max_ = 1.000	*l* = −11→13
4788 measured reflections	

### Refinement

**Table:**

Refinement on *F*^2^	Primary atom site location: heavy-atom method
Least-squares matrix: full	Secondary atom site location: difference Fourier map
*R*[*F*^2^ > 2σ(*F*^2^)] = 0.034	Hydrogen site location: inferred from neighbouring sites
*wR*(*F*^2^) = 0.091	H atoms treated by a mixture of independent and constrained refinement
*S* = 1.02	*w* = 1/[σ^2^(*F*_o_^2^) + (0.0367*P*)^2^] where *P* = (*F*_o_^2^ + 2*F*_c_^2^)/3
1861 reflections	(Δ/σ)_max_ < 0.001
138 parameters	Δρ_max_ = 1.42 e Å^−^^3^
4 restraints	Δρ_min_ = −1.52 e Å^−^^3^

### Special details

**Table:**

Geometry. All e.s.d.'s (except the e.s.d. in the dihedral angle between two l.s. planes) are estimated using the full covariance matrix. The cell e.s.d.'s are taken into account individually in the estimation of e.s.d.'s in distances, angles and torsion angles; correlations between e.s.d.'s in cell parameters are only used when they are defined by crystal symmetry. An approximate (isotropic) treatment of cell e.s.d.'s is used for estimating e.s.d.'s involving l.s. planes.
Refinement. Refinement of *F*^2^ against ALL reflections. The weighted *R*-factor *wR* and goodness of fit *S* are based on *F*^2^, conventional *R*-factors *R* are based on *F*, with *F* set to zero for negative *F*^2^. The threshold expression of *F*^2^ > 2σ(*F*^2^) is used only for calculating *R*-factors(gt) *etc*. and is not relevant to the choice of reflections for refinement. *R*-factors based on *F*^2^ are statistically about twice as large as those based on *F*, and *R*- factors based on ALL data will be even larger.

### Fractional atomic coordinates and isotropic or equivalent isotropic displacement parameters (Å^2^)

**Table:**

	*x*	*y*	*z*	*U*_iso_*/*U*_eq_	
Pt1	0.7500	0.7500	0.5000	0.02646 (16)	
Co1	0.5000	0.75946 (13)	0.7500	0.0265 (3)	
N1	0.6140 (4)	0.7638 (6)	0.6711 (6)	0.0350 (16)	
C1	0.6649 (5)	0.7601 (6)	0.6125 (6)	0.0281 (17)	
O1	0.5000	0.9327 (7)	0.7500	0.043 (2)	
H1	0.460 (3)	0.9754 (17)	0.715 (7)	0.064*	
N2	0.6401 (4)	0.9291 (7)	0.3338 (6)	0.0447 (18)	
C2	0.6790 (5)	0.8641 (8)	0.3938 (7)	0.0365 (19)	
N4	0.5000	0.5778 (7)	0.7500	0.0274 (19)	
C7	0.5912 (4)	0.6000 (7)	0.9333 (7)	0.0304 (17)	
C9	0.5471 (5)	0.4041 (7)	0.8438 (7)	0.039 (2)	
H9	0.5785	0.3641	0.9070	0.047*	
C4	0.6723 (5)	0.7523 (8)	1.0967 (7)	0.044 (2)	
H4	0.6983	0.8060	1.1518	0.052*	
N3	0.5766 (4)	0.7150 (6)	0.9158 (5)	0.0310 (15)	
C6	0.6475 (4)	0.5594 (7)	1.0321 (6)	0.038 (2)	
H6	0.6570	0.4802	1.0426	0.046*	
C8	0.5467 (5)	0.5248 (7)	0.8418 (7)	0.037 (2)	
C5	0.6885 (5)	0.6369 (9)	1.1133 (7)	0.046 (2)	
H5	0.7267	0.6110	1.1786	0.055*	
C3	0.6164 (5)	0.7895 (8)	0.9965 (7)	0.040 (2)	
H3	0.6065	0.8686	0.9854	0.047*	
C10	0.5000	0.3445 (11)	0.7500	0.040 (3)	
H10	0.5000	0.2637	0.7500	0.047*	

### Atomic displacement parameters (Å^2^)

**Table:**

	*U*^11^	*U*^22^	*U*^33^	*U*^12^	*U*^13^	*U*^23^
Pt1	0.0223 (2)	0.0295 (3)	0.0281 (2)	0.00024 (18)	0.00559 (15)	−0.0007 (2)
Co1	0.0227 (7)	0.0306 (9)	0.0266 (7)	0.000	0.0054 (5)	0.000
N1	0.030 (4)	0.042 (5)	0.034 (3)	−0.004 (3)	0.008 (3)	−0.008 (3)
C1	0.023 (4)	0.032 (5)	0.029 (4)	0.000 (3)	0.002 (3)	−0.008 (4)
O1	0.028 (5)	0.032 (5)	0.064 (6)	0.000	−0.005 (4)	0.000
N2	0.038 (4)	0.048 (5)	0.047 (4)	0.009 (4)	0.002 (3)	0.000 (4)
C2	0.032 (4)	0.040 (5)	0.036 (4)	−0.004 (4)	0.000 (3)	0.000 (4)
N4	0.028 (5)	0.020 (5)	0.035 (5)	0.000	0.007 (4)	0.000
C7	0.026 (4)	0.030 (5)	0.035 (4)	0.007 (3)	0.008 (3)	0.004 (4)
C9	0.034 (4)	0.036 (5)	0.044 (5)	0.002 (4)	−0.003 (3)	0.004 (4)
C4	0.044 (5)	0.056 (6)	0.030 (4)	−0.003 (5)	0.004 (3)	−0.010 (4)
N3	0.031 (4)	0.034 (4)	0.028 (3)	0.002 (3)	0.006 (3)	0.000 (3)
C6	0.037 (5)	0.036 (5)	0.038 (5)	0.007 (4)	−0.001 (3)	0.008 (4)
C8	0.029 (4)	0.042 (5)	0.039 (5)	0.004 (4)	0.005 (3)	0.004 (4)
C5	0.041 (5)	0.056 (6)	0.037 (5)	0.009 (4)	−0.005 (4)	0.002 (5)
C3	0.045 (5)	0.039 (5)	0.035 (5)	0.003 (4)	0.008 (4)	0.003 (4)
C10	0.032 (6)	0.026 (7)	0.060 (8)	0.000	0.007 (5)	0.000

### Geometric parameters (Å, º)

**Table:**

Pt1—C1	1.997 (8)	C7—C6	1.400 (10)
Pt1—C1^i^	1.997 (8)	C7—C8	1.450 (11)
Pt1—C2	2.005 (8)	C9—H9	0.9300
Pt1—C2^i^	2.005 (8)	C9—C8	1.391 (11)
Co1—N1	2.128 (6)	C9—C10	1.384 (10)
Co1—N1^ii^	2.128 (6)	C4—H4	0.9300
Co1—O1	1.995 (8)	C4—C5	1.360 (12)
Co1—N4	2.092 (9)	C4—C3	1.395 (11)
Co1—N3^ii^	2.139 (6)	N3—C3	1.340 (10)
Co1—N3	2.139 (6)	C6—H6	0.9300
N1—C1	1.122 (10)	C6—C5	1.372 (11)
O1—H1	0.849 (7)	C5—H5	0.9300
N2—C2	1.128 (10)	C3—H3	0.9300
N4—C8	1.329 (8)	C10—C9^ii^	1.384 (9)
N4—C8^ii^	1.329 (8)	C10—H10	0.9300
C7—N3	1.353 (9)		
			
C1—Pt1—C1^i^	179.999 (2)	N3—C7—C6	121.1 (7)
C1—Pt1—C2^i^	90.9 (3)	N3—C7—C8	115.3 (7)
C1^i^—Pt1—C2^i^	89.1 (3)	C6—C7—C8	123.6 (8)
C1^i^—Pt1—C2	90.9 (3)	C8—C9—H9	120.6
C1—Pt1—C2	89.1 (3)	C10—C9—H9	120.6
C2^i^—Pt1—C2	179.998 (1)	C10—C9—C8	118.8 (8)
N1—Co1—N1^ii^	177.3 (4)	C5—C4—H4	120.2
N1—Co1—N3^ii^	91.5 (2)	C5—C4—C3	119.6 (8)
N1—Co1—N3	89.1 (2)	C3—C4—H4	120.2
N1^ii^—Co1—N3	91.5 (2)	C7—N3—Co1	115.1 (5)
N1^ii^—Co1—N3^ii^	89.1 (2)	C3—N3—Co1	126.3 (6)
O1—Co1—N1^ii^	88.67 (18)	C3—N3—C7	118.3 (7)
O1—Co1—N1	88.67 (19)	C7—C6—H6	120.1
O1—Co1—N4	180.000 (3)	C5—C6—C7	119.8 (8)
O1—Co1—N3^ii^	103.85 (18)	C5—C6—H6	120.1
O1—Co1—N3	103.85 (18)	N4—C8—C7	115.9 (8)
N4—Co1—N1	91.33 (18)	N4—C8—C9	118.3 (8)
N4—Co1—N1^ii^	91.33 (19)	C9—C8—C7	125.8 (7)
N4—Co1—N3^ii^	76.15 (18)	C4—C5—C6	118.9 (8)
N4—Co1—N3	76.15 (18)	C4—C5—H5	120.6
N3—Co1—N3^ii^	152.3 (4)	C6—C5—H5	120.6
C1—N1—Co1	168.1 (6)	C4—C3—H3	118.9
N1—C1—Pt1	176.4 (6)	N3—C3—C4	122.2 (8)
Co1—O1—H1	125.4 (14)	N3—C3—H3	118.9
N2—C2—Pt1	179.0 (8)	C9^ii^—C10—C9	120.5 (12)
C8—N4—Co1	117.4 (5)	C9—C10—H10	119.7
C8^ii^—N4—Co1	117.4 (5)	C9^ii^—C10—H10	119.7
C8^ii^—N4—C8	125.3 (10)		
			
Co1—N4—C8—C7	1.2 (7)	N3^ii^—Co1—N4—C8^ii^	−2.9 (4)
Co1—N4—C8—C9	179.6 (5)	N3—Co1—N4—C8	−2.9 (4)
Co1—N3—C3—C4	−173.3 (6)	N3—Co1—N4—C8^ii^	177.1 (4)
N1—Co1—N4—C8	85.8 (4)	N3^ii^—Co1—N3—C7	4.3 (5)
N1—Co1—N4—C8^ii^	−94.2 (4)	N3^ii^—Co1—N3—C3	178.1 (6)
N1^ii^—Co1—N4—C8^ii^	85.8 (4)	N3—C7—C6—C5	0.3 (11)
N1^ii^—Co1—N4—C8	−94.2 (4)	N3—C7—C8—N4	2.6 (10)
N1^ii^—Co1—N3—C7	95.3 (5)	N3—C7—C8—C9	−175.7 (7)
N1—Co1—N3—C7	−87.3 (5)	C6—C7—N3—Co1	173.3 (5)
N1^ii^—Co1—N3—C3	−90.9 (6)	C6—C7—N3—C3	−1.0 (11)
N1—Co1—N3—C3	86.5 (6)	C6—C7—C8—N4	−175.7 (6)
O1—Co1—N1—C1	−107 (3)	C6—C7—C8—C9	6.0 (13)
O1—Co1—N3—C7	−175.7 (5)	C8^ii^—N4—C8—C7	−178.8 (7)
O1—Co1—N3—C3	−1.9 (6)	C8^ii^—N4—C8—C9	−0.4 (5)
N4—Co1—N1—C1	73 (3)	C8—C7—N3—Co1	−5.0 (8)
N4—Co1—N3—C7	4.3 (5)	C8—C7—N3—C3	−179.4 (6)
N4—Co1—N3—C3	178.1 (6)	C8—C7—C6—C5	178.6 (7)
C7—N3—C3—C4	0.4 (11)	C8—C9—C10—C9^ii^	−0.4 (5)
C7—C6—C5—C4	1.0 (12)	C5—C4—C3—N3	1.0 (12)
N3—Co1—N1—C1	149 (3)	C3—C4—C5—C6	−1.6 (12)
N3^ii^—Co1—N1—C1	−3 (3)	C10—C9—C8—N4	0.7 (10)
N3^ii^—Co1—N4—C8	177.1 (4)	C10—C9—C8—C7	179.0 (6)

Symmetry codes: (i) −*x*+3/2, −*y*+3/2, −*z*+1; (ii) −*x*+1, *y*, −*z*+3/2.

### Hydrogen-bond geometry (Å, º)

**Table:**

*D*—H···*A*	*D*—H	H···*A*	*D*···*A*	*D*—H···*A*
O1—H1···N2^iii^	0.85 (1)	1.93 (2)	2.764 (8)	168 (9)

Symmetry code: (iii) −*x*+1, −*y*+2, −*z*+1.
