# Supplementary figures and images for: Crystal structure of catena-poly[[aqua(2,2′:6′,2′′-terpyridine-κ3 N,N′,N′′)cobalt(II)]-μ-cyanido-κ2 N:C-[dicyanidoplatinum(II)]-μ-cyanido-κ2 C:N]
Source: Acta Crystallogr Sect E Struct Rep Online. 2014 Aug 6;70(Pt 9):m322–3. doi: 10.1107/S1600536814017425 (PMC4186089; doi:10.1107/S1600536814017425)

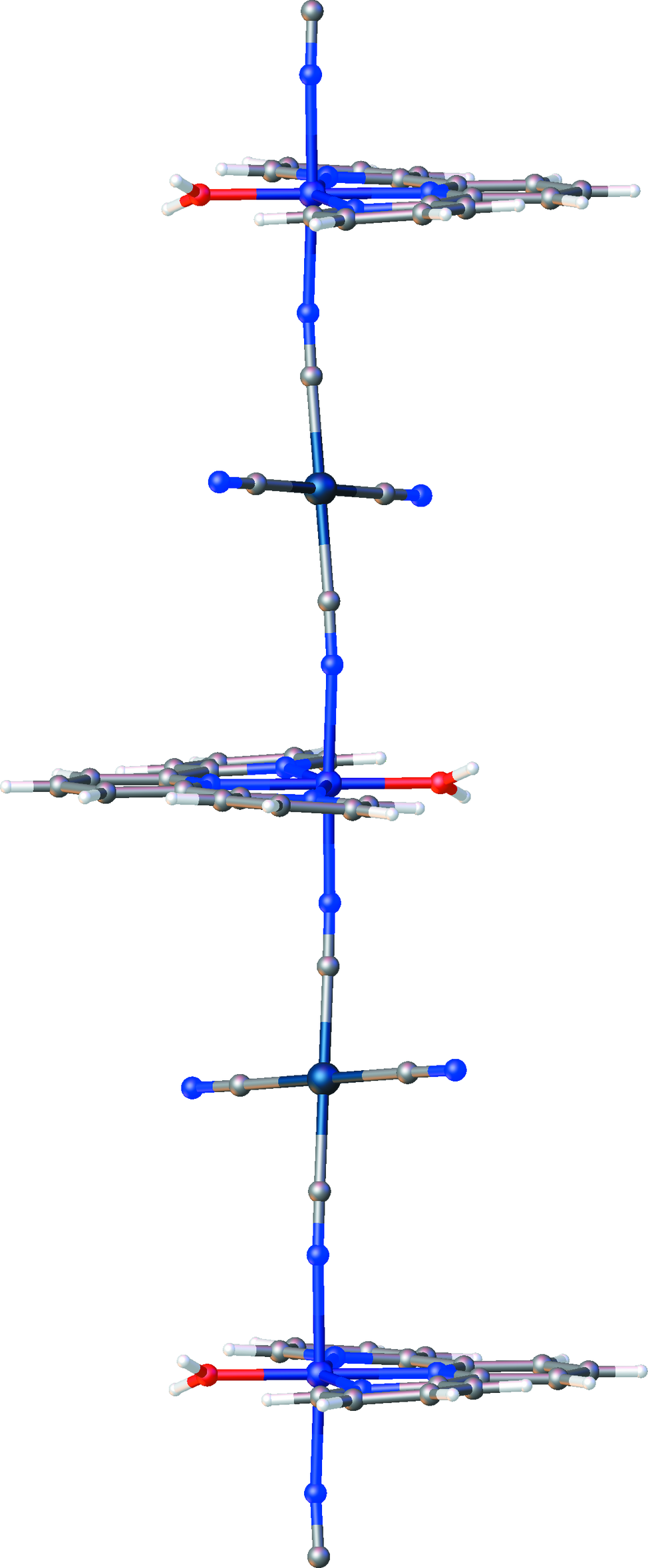

Supplement: Supplementary file 3 [file e-70-0m322-fig1.tif]

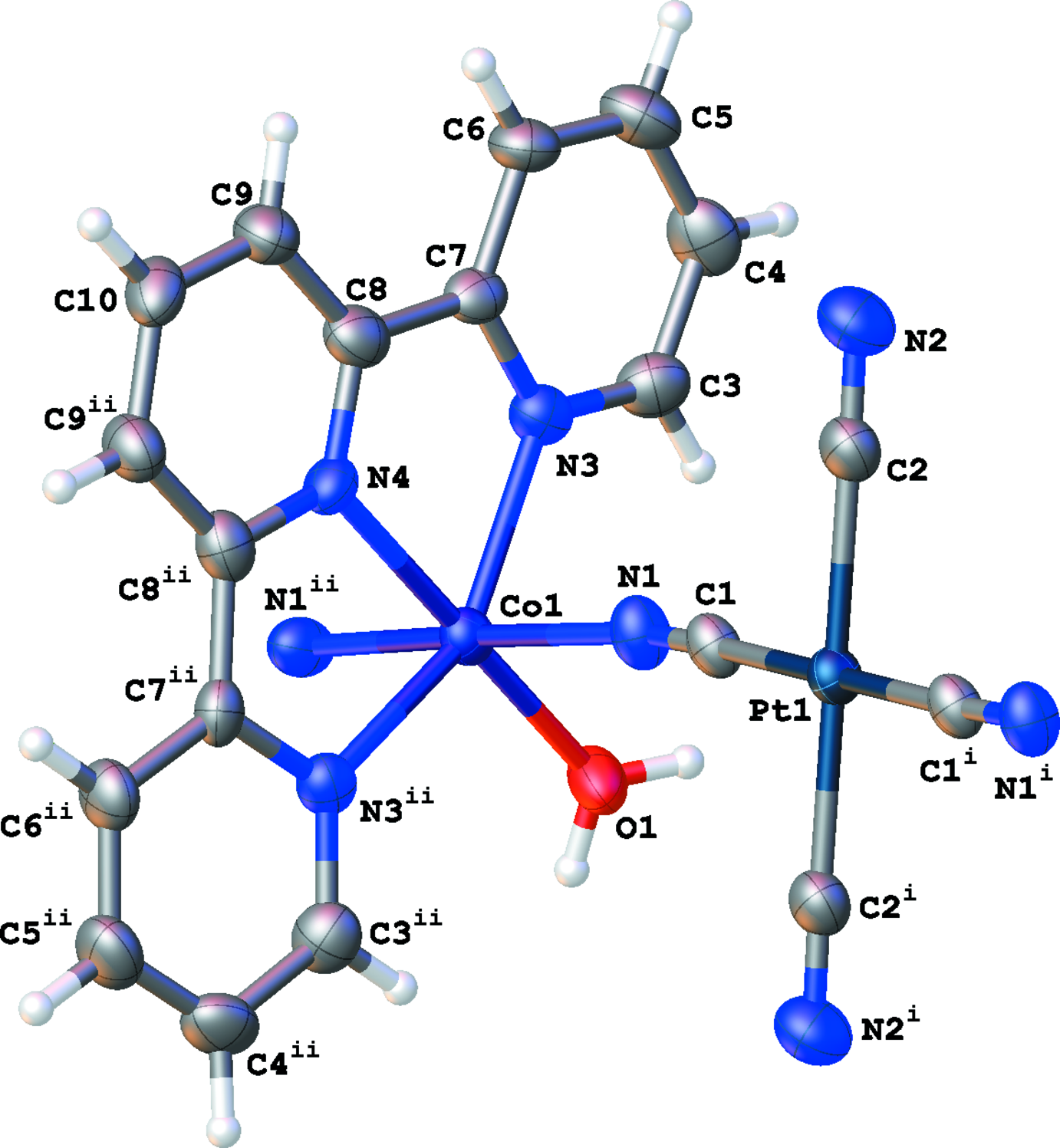

Supplement: Supplementary file 4 [file e-70-0m322-fig2.tif]
